# Orders between Channels and Implications for Partial Information Decomposition

**DOI:** 10.3390/e25070975

**Published:** 2023-06-25

**Authors:** André F. C. Gomes, Mário A. T. Figueiredo

**Affiliations:** Instituto de Telecomunicações and LUMLIS (Lisbon ELLIS Unit), Instituto Superior Técnico, Universidade de Lisboa, 1049-001 Lisboa, Portugal; mario.figueiredo@tecnico.ulisboa.pt

**Keywords:** information theory, partial information decomposition, channel preorders, intersection information, shared information, redundancy

## Abstract

The partial information decomposition (PID) framework is concerned with decomposing the information that a set of random variables has with respect to a target variable into three types of components: redundant, synergistic, and unique. Classical information theory alone does not provide a unique way to decompose information in this manner, and additional assumptions have to be made. Recently, Kolchinsky proposed a new general axiomatic approach to obtain measures of redundant information based on choosing an order relation between information sources (equivalently, order between communication channels). In this paper, we exploit this approach to introduce three new measures of redundant information (and the resulting decompositions) based on well-known preorders between channels, contributing to the enrichment of the PID landscape. We relate the new decompositions to existing ones, study several of their properties, and provide examples illustrating their novelty. As a side result, we prove that any preorder that satisfies Kolchinsky’s axioms yields a decomposition that meets the axioms originally introduced by Williams and Beer when they first proposed PID.

## 1. Introduction

Williams and Beer [1] proposed the partial information decomposition (PID) framework as a way to characterize or analyze the information that a set of random variables (often called *sources*) has about another variable (referred to as the *target*). PID is a useful tool for gathering insights and analyzing the way information is stored, modified, and transmitted within complex systems [2,3]. It has found applications in areas such as cryptography [4] and neuroscience [5,6], with many other potential use cases, such as in understanding how information flows function in gene regulatory networks [7], neural coding [8], financial markets [9], and network design [10].

Consider the simplest case, that of a three-variable joint distribution $p(y_{1},y_{2},t)$ describing three random variables: two sources $Y_{1}$ and $Y_{2}$ and a target *T*. Notice that despite the names *sources* and *target*, there is no directionality assumption, either causal or otherwise. The goal of PID is to *decompose* the information that $Y=(Y_{1},Y_{2})$ has about *T* into the sum of four non-negative quantities: the information that is present in both $Y_{1}$ and $Y_{2}$, known as *redundant* information *R*; the information that only $Y_{1}$ (respectively, $Y_{2}$) has about *T*, known as *unique* information $U_{1}$ (respectively, $U_{2}$); and the *synergistic* information *S* that is present in the pair $\left(Y_{1},Y_{2}\right)$, and is not present in either $Y_{1}$ or $Y_{2}$ alone. That is, in this case with two variables, the goal is to write

(1) $$I\left(T;Y\right)=R+U_{1}+U_{2}+S,$$

where I(T;Y) is the mutual information between *T* and *Y* [11]. In this paper, mutual information is always assumed to refer to Shannon’s mutual information, which for two discrete variables X∈X and Z∈Z is provided by

$$I\left(X;Z\right)=\sum_{x\in X}\sum_{z\in Z}p\left(x,z\right)\log\frac{p(x,z)}{p(x)\;p(z)},$$

and satisfies the following well-known fundamental properties: I(X;Z)≥0 and I(X;Z)=0⇔X⊥Z (*X* and *Z* are independent) [11].

Because unique information and redundancy satisfy the relationship $U_{i}=I\left(T;Y_{i}\right)-R$ (for i∈{1,2}), it turns out that defining how to compute one of these quantities (*R*, $U_{i}$, or *S*) is enough to fully determine the others [1]. As the number of variables grows, the number of terms appearing in the PID of I(T;Y) grows super-exponentially [12]. Williams and Beer [1] suggested a set of axioms that a measure of redundancy should satisfy and proposed a measure of their own. These axioms have become known as the Williams–Beer axioms, although the measure they proposed has subsequently been criticized for not capturing informational content, only information size [13].

Spawned by their initial work, other measures and axioms for information decomposition have been introduced; see, for example, the work by Bertschinger et al. [14], Griffith and Koch [15], and James et al. [16]. There is no consensus about what axioms any measure should satisfy or whether a given measure is *capturing the information* that it should capture other than the Williams–Beer axioms. Today, debate continues about which axioms a measure of redundant information ought to satisfy, and there is no general agreement on what makes for an appropriate PID [16,17,18,19,20].

Recently, Kolchinsky [21] suggested a new general approach to defining measures of redundant information, known as *intersection information* (II), the designation that we adopt hereinafter. The core of this approach is the choice of an order relation between information sources (random variables), which allows two sources to be compared in terms of how informative they are with respect to the target variable.

In this work, we use previously studied preorders between communication channels, which correspond to preorders between the corresponding output variables in terms of information content with respect to the input. Following Kolchinsky’s approach, we show that these orders lead to the definition of new II measures. The rest of the paper is organized as follows. In Section 2 and Section 3, we review Kolchinsky’s definition of an II measure and the *degradation* order. In Section 4, we describe a number of preorders between channels then, based on the work by Korner and Marton [22] and Américo et al. [23], we derive the resulting II measures and study of their properties. Section 5 presents comments on the optimization problems involved in computation of the proposed measures. In Section 6, we explore the relationships between the new II measures and previous PID approaches, then apply the proposed II measures to several famous PID problems. Section 7 concludes the paper by pointing out suggestions for future work.

## 2. Kolchinsky’s Axioms and Intersection Information

Consider a set of *n* discrete random variables $Y_{1}\in Y_{1},\ldots ,Y_{n}\in Y_{n}$, called the source variables, and let T∈T be the target variable (also discrete), with a joint distribution (probability mass function) $p(y_{1},\ldots ,y_{n},t)$. Let ⪯ denote some preorder between random variables that satisfies the following axioms, herein referred to as *Kolchinsky’s axioms* [21]:

**(i)**  Monotonicity of *mutual information* w.r.t. *T*: $Y_{i}⪯Y_{j}\;\Rightarrow \;I\left(Y_{i};T\right)\leq I\left(Y_{j};T\right)$.
**(ii)**  Reflexivity: $Y_{i}⪯Y_{i}$ for all $Y_{i}$.
**(iii)**  For any $Y_{i}$, $C⪯Y_{i}⪯\left(Y_{1},\ldots ,Y_{n}\right)$, where C∈C is any variable taking a constant value with probability one, i.e., with a distribution that is a delta function or such that C is a singleton.

Kolchinsky [21] showed that such an order can be used to define an II measure via

(2) $$I_{\cap }\left(Y_{1},\ldots ,Y_{n}\to T\right):=\underset{Q:\;Q⪯Y_{i},\;i\in \left\{1,..,n\right\}}{\sup}I\left(Q;T\right),$$

and we now show that this implies that the II measure in (2) satisfies the Williams–Beer axioms [1,2], establishing a strong connection between these formulations. Before stating and proving this result, we first recall the Williams–Beer axioms [2], where the definition of a source $A_{i}$ is that of a set of random variables, e.g., $A_{1}=\left\{X_{1},X_{2}\right\}$.

**Definition** **1.** *Let $A_{1},\ldots ,A_{r}$ be an arbitrary number of r≥2 sources. An intersection information measure $I_{\cap }$ is said to satisfy the Williams-Beer axioms if it satisfies the following:*

*1.*  *Symmetry: $I_{\cap }$ is symmetric in the $A_{i}$s*.
*2.*  *Self-redundancy: $I_{\cap }\left(A_{i}\right)=I\left(A_{i};T\right)$.*
*3.*  *Monotonicity: $I_{\cap }\left(A_{1},\ldots ,A_{r-1},A_{r}\right)\leq I_{\cap }\left(A_{1},\ldots ,A_{r-1}\right)$.*
*4.*  *Equality for Monotonicity: If $A_{r-1}\subseteq A_{r}$, then $I_{\cap }\left(A_{1},\ldots ,A_{r-1},A_{r}\right)=I_{\cap }\left(A_{1},\ldots ,A_{r-1}\right)$.*

**Theorem** **1.** *Let ⪯ be some preorder that satisfies Kolchinsky’s axioms, and define its corresponding II measure as in (2). Then, the corresponding II measure satisfies the Williams–Beer axioms.*

**Proof.**  Symmetry and monotonicity follow trivially given the form of (2) (the definition of the supremum and restriction set). Self-redundancy follows from the reflexivity of the preorder and monotonicity of mutual information. Now, suppose $A_{r-1}\subseteq A_{r}$, and let *Q* be a solution of $I_{\cap }\left(A_{1},\ldots ,A_{r-1}\right)$, implying that $Q⪯A_{r-1}$. Now, because $A_{r-1}\subseteq A_{r}$, the third Kolchinsky axiom and transitivity of the preorder ⪯ guarantee that $Q⪯\;A_{r-1}⪯A_{r}$, meaning that *Q* is an admissible point of $I_{\cap }\left(A_{1},\ldots ,A_{r}\right)$. Therefore, $I_{\cap }\left(A_{1},\ldots ,A_{r-1},A_{r}\right)\geq I_{\cap }\left(A_{1},\ldots ,A_{r-1}\right)$ and monotonicity guarantees that $I_{\cap }\left(A_{1},\ldots ,A_{r-1},A_{r}\right)=I_{\cap }\left(A_{1},\ldots ,A_{r-1}\right)$.    □

In conclusion, every preorder relation that satisfies the set of axioms introduced by Kolchinsky [21] yields a valid II measure, in the sense that the measure satisfies the Williams–Beer axioms. Having a *more informative* relation ⪯ allows us to draw conclusions about information flowing from different sources, and allows for the construction of PID measures that are well-defined for more than two sources. In the following, we omit “→T” from the notation unless we need to explicitly refer to it, with the understanding that the target variable is always some arbitrary discrete random variable *T*.

## 3. Channels and the Degradation/Blackwell Order

In an information-theoretical perspective, given two discrete random variables X∈X and Z∈Z, the corresponding conditional distribution p(z|x) corresponds to a discrete memoryless channel with a channel matrix *K* such that K[x,z]=p(z|x) [11]. This matrix is row-stochastic, i.e., K[x,z]≥0 for any x∈X and z∈Z, and $\sum_{z\in Z}K\left[x,z\right]=1$.

The comparison of different channels (equivalently, different stochastic matrices) is an object of study with many applications in different fields [24]. Such investigations address order relations between channels and their properties. One such order, named the *degradation order* (or *Blackwell order*) and defined next, was used by Kolchinsky to obtain a particular II measure [21].

Consider the distribution $p(y_{1},\ldots ,y_{n},t)$ and the channels $K^{\left(i\right)}$ between *T* and each $Y_{i}$, that is, $K^{\left(i\right)}$ is a $\left|T|\times \right|Y_{i}|$ row-stochastic matrix with the conditional distribution $p(y_{i}|t)$.

**Definition** **2.** *We say that channel $K^{\left(i\right)}$ is a* degradation *of channel $K^{\left(j\right)}$, and write $K^{\left(i\right)}{⪯}_{d}K^{\left(j\right)}$ or $Y_{i}{⪯}_{d}Y_{j}$, if there exists a channel $K^{U}$ from $Y_{j}$ to $Y_{i}$, i.e., a $|Y_{j}\left|\times \right|Y_{i}|$ row-stochastic matrix, such that $K^{\left(i\right)}=K^{\left(j\right)}K^{U}$.*

Intuitively, consider two agents, one with access to $Y_{i}$ and the other with access to $Y_{j}$. The agent with access to $Y_{j}$ has at least as much information about *T* as the one with access to $Y_{i}$, as it has access to channel $K^{U}$, which permits sampling from $Y_{i}$ conditionally on $Y_{j}$ [19]. Blackwell [25] showed that this is equivalent to saying that, for whatever decision game where the goal is to predict *T* and for whatever utility function, the agent with access to $Y_{i}$ cannot do better on average than the agent with access to $Y_{j}$.

Based on the degradation/Blackwell order, Kolchinsky [21] introduced the *degradation II measure* by plugging the “${⪯}_{d}$” order into (2):

(3) $$I_{\cap }^{d}\left(Y_{1},\ldots ,Y_{n}\right):=\underset{Q:\;Q{⪯}_{d}Y_{i},\;i\in \left\{1,..,n\right\}}{\sup}I\left(Q;T\right).$$

As noted by Kolchinsky [21], this II measure has the following operational interpretation. Supposing that n=2 and considering two agents, 1 and 2, with access to variables $Y_{1}$ and $Y_{2}$, respectively, $I_{\cap }^{d}\left(Y_{1},Y_{2}\right)$ is the maximum information that agent 1 (respectively 2) can have with respect to *T* without being able to do better than agent 2 (respectively 1) on any decision problem that involves guessing *T*. That is, the degradation II measure quantifies the existence of a dominating strategy for any guessing game.

## 4. Other Orders and Corresponding II Measures

### 4.1. The “Less Noisy” Order

Korner and Marton [22] introduced and studied preorders between channels with the same input. We follow most of their definitions, and change others when appropriate. We interchangeably write $Y_{1}⪯Y_{2}$ to mean $K^{\left(1\right)}⪯K^{\left(2\right)}$, where $K^{\left(1\right)}$ and $K^{\left(2\right)}$ are the channel matrices defined above.

Before introducing the next channel order, we need to review the notion of Markov chains [11]. We can say that three random variables $X_{1}$, $X_{2}$, and $X_{3}$ form a Markov chain, for which we write $X_{1}\to X_{2}\to X_{3}$, if the following equality holds: $p\left(x_{1},x_{3}|x_{2}\right)=p\left(x_{1}|x_{2}\right)\;p\left(x_{3}|x_{2}\right)$, i.e., if $X_{1}$ and $X_{3}$ are conditionally independent given $X_{2}$. Of course, $X_{1}\to X_{2}\to X_{3}$ if and only if $X_{3}\to X_{2}\to X_{1}$.

**Definition** **3.** *We say that channel $K^{\left(2\right)}$ is* less noisy *than channel $K^{\left(1\right)}$, and write $K^{\left(1\right)}{⪯}_{ln}K^{\left(2\right)}$, if for any discrete random variable U with finite support (such that both $U\to T\to Y_{1}$ and $U\to T\to Y_{2}$ hold) we have $I\left(U;Y_{1}\right)\leq I\left(U;Y_{2}\right)$.*

The *less noisy* order has been primarily used in network information theory to study the problems of the capacity regions of broadcast channels [26] and the secrecy capacity of wiretap and eavesdrop channels [27]. The secrecy capacity ($C_{S}$) is the maximum rate at which information can be transmitted over a communication channel while keeping the communication secure from eavesdroppers, that is, having zero information leakage [28,29]. It has been shown that $C_{S}>0$ unless $K^{\left(2\right)}{⪯}_{ln}K^{\left(1\right)}$, where $C_{S}$ is the secrecy capacity of the Wyner wiretap channel, with $K^{\left(2\right)}$ as the main channel and $K^{\left(1\right)}$ as the eavesdropper channel ([27], Corollary 17.11).

Plugging the *less noisy* order ${⪯}_{ln}$ into (2) yields a new II measure

(4) $$I_{\cap }^{ln}\left(Y_{1},\ldots ,Y_{n}\right):=\underset{Q:\;Q{⪯}_{ln}Y_{i},\;i\in \left\{1,..,n\right\}}{\sup}I\left(Q;T\right).$$

Intuitively, $I_{\cap }^{ln}\left(Y_{1},\ldots ,Y_{n}\right)$ is the most information that a channel $K^{Q}$ can have about *T* such that it is *less noisy* than any other channel $K^{\left(i\right)},i=1,\ldots ,n$, that is, a channel that leads to a positive secrecy capacity, as compared to any other channel $K^{\left(i\right)}$.

### 4.2. The “More Capable” Order

The next order we consider, termed “*more capable*”, has been used in calculating the capacity region of broadcast channels [30] and to help determine whether one system is more secure than another [31]; see the book by Cohen et al. [24] for more applications of the *degradation, less noisy*, and *more capable* orders.

**Definition** **4.** *We say that channel $K^{\left(2\right)}$ is* more capable *than $K^{\left(1\right)}$, and write $K^{\left(1\right)}\;{⪯}_{mc}\;K^{\left(2\right)}$, if for any distribution p(t) we have $I\left(T;Y_{1}\right)\leq I\left(T;Y_{2}\right)$.*

Inserting the “*more capable*” order into (2) leads to

(5) $$I_{\cap }^{mc}\left(Y_{1},\ldots ,Y_{n}\right):=\underset{Q:\;Q{⪯}_{mc}Y_{i},\;i\in \left\{1,..,n\right\}}{\sup}I\left(Q;T\right),$$

that is, $I_{\cap }^{mc}\left(Y_{1},\ldots ,Y_{n}\right)$ is the information that the ‘largest’ (in the more capable sense), though no larger than any $Y_{i}$, that the random variable *Q* has with respect to *T*. Whereas under the degradation order it is guaranteed that agent 2 will make better decisions if $Y_{1}{⪯}_{d}Y_{2}$ for whatever decision game, on average, under the “more capable“ order, such a guarantee is not available. However, we do have a guarantee that, if $Y_{1}{⪯}_{mc}Y_{2}$, then for a given distribution p(t) we know that agent 2 always has more information about *T* than agent 1. This has an interventional approach meaning; if we intervene on variable *T* by changing its distribution p(t) in whichever way we see fit, we have $I\left(Y_{1};T\right)\leq I\left(Y_{2};T\right)$ (assuming that the distribution $p(Y_{1},\ldots ,Y_{n},T)$ can be modeled as a set of channels from *T* to each $Y_{i}$); that is to say, $I_{\cap }^{mc}\left(Y_{1},\ldots ,Y_{n}\right)$ is the highest information that a channel $K^{Q}$ can have about *T* such that for any change in p(t), $K^{Q}$ knows less about *T* than any $Y_{i},i=1,\ldots ,n$. Because PID is concerned with decomposing a distribution that has fixed p(t), the “more capable” measure is concerned with the mechanism by which *T* generates $Y_{1},\ldots ,Y_{n}$ for any p(t), and is not concerned with the specific distribution p(t) yielded by $p(Y_{1},\ldots ,Y_{n},T)$.

For the sake of completeness, we could additionally study the II measure that would result from the capacity order. Recall that the capacity of the channel from a variable *X* to another variable *Z*, which is only a function of the conditional distribution p(z|x), is defined as [11]

(6) $$C=\max_{p(x)}I\left(X;Z\right).$$

**Definition** **5.** *We can write $W{⪯}_{c}V$ if the capacity of V is at least as large as the capacity of W.*

Even though it is clear that $W{⪯}_{mc}V\;\Rightarrow \;W{⪯}_{c}V$, the ${⪯}_{c}$ order does not comply with the first of Kolchinsky’s axioms, as the definition of capacity involves the choice of a particular marginal that achieves the maximum in (6), which may not coincide with the marginal corresponding to $p(y_{1},\ldots ,y_{n},t)$. For this reason, we do not define an II measure based on it.

### 4.3. The “Degradation/Supermodularity” Order

In order to introduce the last II measure, we follow the work and notation of Américo et al. [23]. Given two real vectors *r* and *s* with dimension *n*, let $r\vee s:=(\max\left(r_{1},s_{1}\right),$$\ldots ,\max(r_{n},s_{n}))$ and $r\wedge s:=(\min\left(r_{1},s_{1}\right),\ldots ,\min\left(r_{n},s_{n}\right))$. Consider an arbitrary channel *K*, and let $K_{i}$ be its *i*th column. From *K*, we may define a new channel which we construct column by column using the *JoinMeet* operator ${⋄}_{i,j}$. Column *l* of the new channel is defined for i≠j as

$${\left({⋄}_{i,j}K\right)}_{l}=\left\{\begin{array}{cc} K_{i}\vee K_{j}, & if\;l=i\\ K_{i}\wedge K_{j}, & if\;l=j\\ K_{l}, & otherwise \end{array}\right..$$

Américo et al. [23] used this operator to define the two new orders described below. Intuitively, the operator ${⋄}_{i,j}$ makes the rows of the channel matrix more similar to each other by putting all the maxima in column *i* and the minima in column *j* between every pair of elements in columns *i* and *j* of every row. In the following definitions, the *s* stands for supermodularity, a concept we need not introduce in this work.

**Definition** **6.** *We can write $W{⪯}_{s}V$ if there exists a finite collection of tuples $\left(i_{k},j_{k}\right)$ such that $W={⋄}_{i_{1},j_{1}}({⋄}_{i_{2},j_{2}}\left(\ldots \left({⋄}_{i_{m},j_{m}}V\right)\right)$.*

**Definition** **7.** *Ww write $W{⪯}_{ds}V$ if there are m channels $U^{\left(1\right)},\ldots ,U^{\left(m\right)}$ such that $W{⪯}_{0}U^{\left(1\right)}{⪯}_{1}U^{\left(2\right)}{⪯}_{2}\ldots {⪯}_{m-1}U^{\left(m\right)}{⪯}_{m}V$, where each ${⪯}_{i}$ stands for ${⪯}_{d}$ or ${⪯}_{s}$. We call this the* degradation/supermodularity *order*.

Using the “*degradation/supermodularity*” (ds) order, we can define the ds II measure as follows:

(7) $$I_{\cap }^{ds}\left(Y_{1},\ldots ,Y_{n}\right):=\underset{Q:\;Q{⪯}_{ds}Y_{i},\;i\in \left\{1,..,n\right\}}{\sup}I\left(Q;T\right).$$

The *ds* order was recently introduced in the context of *core-concave entropies* [23]. Given a core-concave entropy *H*, the *leakage* about *T* through $Y_{1}$ is defined as $I_{H}\left(T;Y_{1}\right)=H\left(T\right)-H\left(T|Y_{1}\right)$. In this work, we are mainly concerned with the Shannon entropy *H*; however, as we elaborate in the future work section at the end of this paper, PID may be applied to other core-concave entropies. Although the operational interpretation of the *ds* order is not yet clear, it has found applications in privacy/security contexts, as well as in finding the most secure deterministic channel (under certain constraints) [23].

### 4.4. Relations between Orders

**Figure 1:**
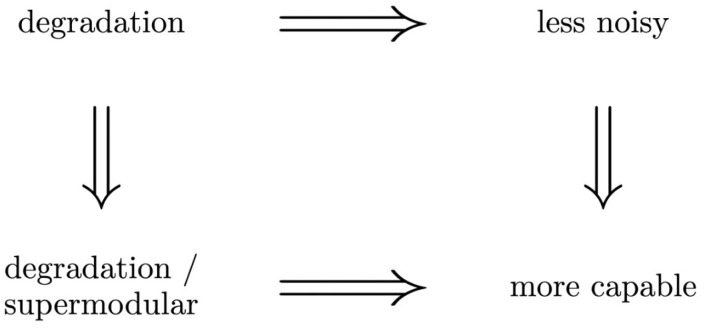
Implications satisfied by the orders. The reverse implications do not hold in general.

Korner and Marton [22] proved that $W{⪯}_{d}V\Rightarrow W{⪯}_{ln}V\Rightarrow W{⪯}_{mc}V$, and provided examples to show that the reverse implications do not hold in general. As Américo et al. [23] note, the degradation (${⪯}_{d}$), supermodularity (${⪯}_{s}$), and degradation/ supermodularity (${⪯}_{ds}$) orders are *structural orders*, in the sense that they only depend on the conditional probabilities that are defined by each channel. On the other hand, the *less noisy* and *more capable* orders are concerned with information measures resulting from different distributions. It is trivial to see (directly from the definition) that the degradation order implies the degradation/supermodular order. In turn, Américo et al. [23] showed that the degradation/supermodular order implies the more capable order. This set of implications is schematically depicted in Figure 1.

For any set of variables $Y_{1},\ldots ,Y_{n},T$, these relations between the orders imply, via the corresponding definitions, that

(8) $$\begin{array}{c} I_{\cap }^{d}\left(Y_{1},\ldots ,Y_{n}\right)\leq I_{\cap }^{ln}\left(Y_{1},\ldots ,Y_{n}\right)\leq I_{\cap }^{mc}\left(Y_{1},\ldots ,Y_{n}\right) \end{array}$$

and

(9) $$\begin{array}{c} I_{\cap }^{d}\left(Y_{1},\ldots ,Y_{n}\right)\leq I_{\cap }^{ds}\left(Y_{1},\ldots ,Y_{n}\right)\leq I_{\cap }^{mc}\left(Y_{1},\ldots ,Y_{n}\right). \end{array}$$

These, in turn, imply the following result.

**Theorem** **2.** *The preorders ${⪯}_{ln}$, ${⪯}_{mc}$, and ${⪯}_{ds}$, satisfy Kolchinsky’s axioms.*

**Proof.**  Let i∈{1,…,n}. Because any of the introduced orders implies the *more capable* order, it follows that they all satisfy the axiom of monotonicity of mutual information. Axiom 2 is trivially true, as reflexivity is guaranteed by the definition of preorder. For axiom 3, the rows of a channel corresponding to a variable *C* taking a constant value must all be the same (and yield zero mutual information with any target variable *T*), from which it is clear that any $Y_{i}$ satisfies $C⪯Y_{i}$ for any of the introduced orders per the definition of each order. To see that $Y_{i}⪯Y=\left(Y_{1},\ldots ,Y_{n}\right)$ for the *less noisy* and the *more capable*, recall that for any *U* such that $U\to T\to Y_{i}$ and U→T→Y it is trivial that $I\left(U;Y_{i}\right)\leq I\left(U;Y\right)$; hence, $Y_{i}{⪯}_{ln}Y$. A similar argument can be used to show that $Y_{i}{⪯}_{mc}Y$, as $I\left(T;Y_{i}\right)\leq I\left(T;Y\right)$. Finally, to see that $Y_{i}{⪯}_{ds}\left(Y_{1},\ldots ,Y_{n}\right)$, note that $Y_{i}{⪯}_{d}\left(Y_{1},\ldots ,Y_{n}\right)$ [21]; hence, $Y_{i}{⪯}_{ds}\left(Y_{1},\ldots ,Y_{n}\right)$.    □

## 5. Optimization Problems

We now focus on certain observations around optimization problems involving the introduced II measures. All of these problems seek to maximize I(Q;T) (under different constraints) as a function of the conditional distribution p(q|t), and equivalently with respect to the channel from *T* to *Q*, which we denote as $K^{Q}:=K^{Q|T}$. For fixed p(t), as is the case in PID, I(Q;T) is a convex function of $K^{Q}$ ([11], Theorem 2.7.4). As we will see, the admissible region of all problems is a compact set, and because I(Q;T) is a continuous function of the parameters of $K^{Q}$, the supremum is achieved; thus, we replace sup here with max.

As noted by Kolchinsky [21], the computation of (3) can be rewritten as an optimization problem using auxiliary variables such that it involves only linear constraints, and because the objective function is convex, its maximum is attained at one of the vertices of the admissible region. The computation of the other measures, however, is not as simple, as shown in the following subsections.

### 5.1. The “Less Noisy” Order

To solve (4), we can use one of the necessary and sufficient conditions presented by (Makur and Polyanskiy [26], Theorem 1). For instance, let *V* and *W* be two channels with input *T*, and let $\Delta^{T-1}$ be the probability simplex of the target *T*. Then, $V{⪯}_{ln}W$, if and only if the inequality

(10) $$\chi^{2}\left(p\left(t\right)W||q\left(t\right)W\right)\geq \chi^{2}\left(p\left(t\right)V||q\left(t\right)V\right)$$

holds for any pair of distributions $p\left(t\right),q\left(t\right)\in \Delta^{T-1}$, where $\chi^{2}$ in the above equation denotes the $\chi^{2}$-distance between two vectors. The $\chi^{2}$ distance between two vectors *u* and *v* of dimension *n* is given by $\chi^{2}\left(u||v\right)=\sum_{i=1}^{n}{\left(u_{i}-v_{i}\right)}^{2}/v_{i}$. Notice that p(t)W is the distribution of the output of channel *W* for input distribution p(t); thus, intuitively, the condition in (10) means that the two output distributions of the *less noisy* channel are more different from each other than those of the other channel. Hence, computing $I_{\cap }^{ln}\left(Y_{1},\ldots ,Y_{n}\right)$ can be formulated as solving the problem

$$\begin{array}{cc} \max_{K^{Q}}\; & I(Q;T)\\ s.t. & \;K^{Q}\;is\;a\;stochastic\;matrix,\\ \; & \;\forall p\left(t\right),q\left(t\right)\in \Delta^{T-1},\forall i\in \left\{1,\ldots ,n\right\},\;\chi^{2}(p\left(t\right)K^{\left(i\right)}||q\left(t\right)K^{\left(i\right)})\geq \chi^{2}(p\left(t\right)K^{Q}||q\left(t\right)K^{Q}). \end{array}$$

Although the restriction set is convex, as the $\chi^{2}$-divergence is an *f*-divergence with convex *f* [27], the problem is intractable because we have an infinite (uncountable) number of restrictions. It is possible to construct a set S by taking an arbitrary number of samples *S* of $p\left(t\right)\in \Delta^{T-1}$ to define the problem

(11) $$\begin{array}{cc} \max_{K^{Q}}\; & I(Q;T),\\ s.t. & \;K^{Q}\;is\;a\;stochastic\;matrix,\\ \; & \;\forall p\left(t\right),q\left(t\right)\in S,\forall i\in \left\{1,\ldots ,n\right\},\;\chi^{2}(p\left(t\right)K^{\left(i\right)}||q\left(t\right)K^{\left(i\right)})\geq \chi^{2}(p\left(t\right)K^{Q}||q\left(t\right)K^{Q}). \end{array}$$

The above problem yields an upper bound on $I_{\cap }^{ln}\left(Y_{1},\ldots ,Y_{n}\right)$.

### 5.2. The “More Capable” Order

To compute $I_{\cap }^{mc}\left(Y_{1},\ldots ,Y_{n}\right)$, we can define the problem

(12) $$\begin{array}{cc} \max_{K^{Q}}\; & I(Q;T)\\ s.t. & \;K^{Q}\;is\;a\;stochastic\;matrix,\\ \; & \;\forall p\left(t\right)\in \Delta^{T-1},\forall i\in \left\{1,\ldots ,n\right\},\;I\left(Y_{i};T\right)\geq I\left(Q;T\right), \end{array}$$

which again leads to a convex restriction set, as I(Q;T) is a convex function of $K^{Q}$. We can discretize the problem in the same manner as above to obtain a tractable version

(13) $$\begin{array}{cc} \max_{K^{Q}}\; & I(Q;T)\\ s.t. & \;K^{Q}\;is\;a\;stochastic\;matrix,\\ \; & \;\forall p\left(t\right)\in S,\forall i\in \left\{1,\ldots ,n\right\},\;I\left(Y_{i};T\right)\geq I\left(Q;T\right), \end{array}$$

which again yields an upper bound on $I_{\cap }^{mc}\left(Y_{1},\ldots ,Y_{n}\right)$.

### 5.3. The “Degradation/Supermodularity” Order

The final introduced measure, $I_{\cap }^{ds}\left(Y_{1},\ldots ,Y_{n}\right)$, is provided by

(14) $$\begin{array}{cc} \max_{K^{Q}}\; & I(Q;T)\\ s.t. & \;K^{Q}\;is\;a\;stochastic\;matrix,\\ \; & \;\forall i,K^{Q}{⪯}_{ds}K^{\left(i\right)}. \end{array}$$

To the best of our knowledge, there is currently no known condition to check whether $K^{Q}\;{⪯}_{ds}\;K^{\left(i\right)}$.

## 6. Relation to Existing PID Measures

Griffith et al. [32] introduced a measure of II as

(15) $$\begin{array}{c} I_{\cap }^{◃}\left(Y_{1},\ldots ,Y_{n}\right):=\max_{Q}I\left(Q;T\right),\;\mathrm{such}\;\mathrm{that}\;\forall i\;Q◃Y_{i}, \end{array}$$

with the order relation ◃ defined by A◃B if A=f(B) for some deterministic function *f*, that is, $I_{\cap }^{◃}$ is used to quantify the redundancy as the presence of deterministic relations between the input and target. If *Q* is a solution of (15), then there exist functions $f_{1},\ldots ,f_{n}$ such that $Q=f_{i}\left(Y_{i}\right),i=1,\ldots ,n$, which implies that for all *i* it is the case that $T\to Y_{i}\to Q$ is a Markov chain. Therefore, *Q* is an admissible point of the optimization problem that defines $I_{\cap }^{d}\left(Y_{1},\ldots ,Y_{n}\right)$, and we have $I_{\cap }^{◃}\left(Y_{1},\ldots ,Y_{n}\right)\leq I_{\cap }^{d}\left(Y_{1},\ldots ,Y_{n}\right)$.

Barrett [33] introduced the so-called *minimum mutual information* (MMI) measure of bivariate redundancy as

$$I_{\cap }^{\mathrm{MMI}}\left(Y_{1},Y_{2}\right):=\min\left\{I\left(T;Y_{1}\right),I\left(T;Y_{2}\right)\right\}.$$

It turns out that if $\left(Y_{1},Y_{2}\right)$ is jointly Gaussian and *T* is univariate, then most of the introduced PIDs in the literature are equivalent to this measure [33]. Furthermore, as noted by Kolchinsky [21], it may be generalized to more than two sources:

$$I_{\cap }^{\mathrm{MMI}}\left(Y_{1},\ldots ,Y_{n}\right):=\underset{Q}{\sup}I\left(Q;T\right),\;\mathrm{such}\;\mathrm{that}\;\forall i\;I\left(Q;T\right)\leq I\left(Y_{i};T\right),$$

which allows us to trivially conclude that for any set of variables $Y_{1},\ldots ,Y_{n},T$,

$$I_{\cap }^{◃}\left(Y_{1},\ldots ,Y_{n}\right)\leq I_{\cap }^{d}\left(Y_{1},\ldots ,Y_{n}\right)\leq I_{\cap }^{mc}\left(Y_{1},\ldots ,Y_{n}\right)\leq I_{\cap }^{\mathrm{MMI}}\left(Y_{1},\ldots ,Y_{n}\right).$$

One of the appeals of measures of II as defined by Kolchinsky [21] is that the underlying preorder determines what is intersection (or redundant) information. For example, taking the degradation II measure in the n=2 case, its solution *Q* satisfies $T⊥Q\;|\;Y_{1}$ and $T⊥Q\;|\;Y_{2}$; that is, if either $Y_{1}$ or $Y_{2}$ are known, then *Q* has no additional information about *T*. The same is not necessarily the case for the *less noisy* or the *more capable* II measures, where the solution *Q* may have additional information about *T* even when a source is known. However, the three proposed measures satisfy the property that any solution *Q* of the optimization problem satisfies

$$\forall i\in \left\{1,\ldots ,n\right\},\forall t\in S_{T},I\left(Y_{i};T=t\right)\geq I\left(Q;T=t\right),$$

where $S_{T}$ is the support of *T* and $I(T=t;Y_{i})$ refers to the so-called specific information [1,34]. This means that, independent of the outcome of *T*, *Q* has less specific information about T=t than any source variable $Y_{i}$. This can be seen by noting that any of the introduced orders imply the more capable order. This is not the case, for example, for $I_{\cap }^{\mathrm{MMI}}$, which is arguably one of the reasons why it has been criticized for depending only on the amount of information and not on its content [21]. As mentioned, there is not much consensus as to which properties a measure of II should satisfy. The three proposed measures for partial information decomposition do not satisfy the so-called *Blackwell property* [14,35]:

**Definition** **8.** *An intersection information measure $I_{\cap }\left(Y_{1},Y_{2}\right)$ is said to satisfy the Blackwell property if the equivalence $Y_{1}{⪯}_{d}Y_{2}\Leftrightarrow I_{\cap }\left(Y_{1},Y_{2}\right)=I\left(T;Y_{1}\right)$ holds.*

This definition is equivalent to demanding that $Y_{1}{⪯}_{d}Y_{2}$ if and only if $Y_{1}$ has no unique information about *T*. Although the (⇒) implication holds for the three proposed measures, the reverse implication does not, as shown by specific examples presented by Korner and Marton [22], which we mention below. If we define the “more capable” property by replacing the *degradation* order with the *more capable* order in the original definition of the Blackwell property, then it is clear that measure *k* satisfies the *k* property, with *k* referring to any of the three introduced intersection information measures.

In PID, the *identity property* (IP) has been frequently studied [13]. For this property, let the target *T* be a copy of the source variables, that is, let $T=(Y_{1},Y_{2})$. An II measure $I_{\cap }$ is said to satisfy the IP if

$$I_{\cap }\left(Y_{1},Y_{2}\right)=I\left(Y_{1};Y_{2}\right).$$

Criticism has been levied against this proposal for being too restrictive [16,36]. A less strict property was introduced by [20] under the name *independent identity property* (IIP). If the target *T* is a copy of the input, an II measure is said to satisfy the IIP if

$$I\left(Y_{1};Y_{2}\right)=0\;\;\Rightarrow \;\;I_{\cap }\left(Y_{1},Y_{2}\right)=0.$$

Note that the IIP is implied by the IP, while the reverse does not hold. It turns out that all the introduced measures, as is the case for the degradation II measure, satisfy the IIP and not the IP, as we show later. This can be seen from (8) and (9), as well as from the fact that $I_{\cap }^{mc}\left(Y_{1},Y_{2}\to \left(Y_{1},Y_{2}\right)\right)$ equals 0 if $I(Y_{1};Y_{2})=0$, as we argue now. Consider the distribution where *T* is a copy of $\left(Y_{1},Y_{2}\right)$, as presented in Table 1.

We assume that each of the four events has non-zero probability. In this case, channels $K^{\left(1\right)}$ and $K^{\left(2\right)}$ are provided by

$$K^{\left(1\right)}=\left[\begin{array}{cc} 1 & 0\\ 1 & 0\\ 0 & 1\\ 0 & 1 \end{array}\right],\;K^{\left(2\right)}=\left[\begin{array}{cc} 1 & 0\\ 0 & 1\\ 1 & 0\\ 0 & 1 \end{array}\right].$$

Note that for any distribution $p\left(t\right)=\left[p(0,0),p(0,1),p(1,0),p(1,1)\right]$, if p(1,0)=p(1,1)=0, then $I(T;Y_{1})=0$, which implies that for any such distributions the solution *Q* of (12) must satisfy I(Q;T)=0. Thus, the first and second rows of $K^{Q}$ must be the same. The same is the case for any distribution p(t) with p(0,0)=p(0,1)=0; on the other hand, if p(0,0)=p(1,0)=0 or p(1,1)=p(0,1)=0, then $I(T;Y_{2})=0$, implying that I(Q;T)=0 for such distributions. Hence, $K^{Q}$ must be an arbitrary channel, that is, a channel that satisfies Q⊥T, yielding $I_{\cap }^{mc}\left(Y_{1},Y_{2}\right)=0$.

Now, recall the Gács–Korner *common information* [37], defined as

(16) $$\begin{array}{ccc} C(Y_{1}\wedge Y_{2}):= & \underset{Q}{\sup} & H(Q)\\ \; & s.t. & Q◃Y_{1}\\ \; & \; & Q◃Y_{2} \end{array}$$

We use a similar argument, while slightly changing the notation, to show the following result.

**Theorem** **3.** *Let T=(X,Y) be a copy of the source variables; then, $I_{\cap }^{ln}\left(X,Y\right)=I_{\cap }^{ds}\left(X,Y\right)=I_{\cap }^{mc}\left(X,Y\right)=C\left(X\wedge Y\right)$.*

**Proof.**  As shown by Kolchinsky [21], $I_{\cap }^{d}\left(X,Y\right)=C\left(X\wedge Y\right)$. Thus, (8) implies that $I_{\cap }^{mc}\left(X,Y\right)$≥C(X∧Y). The proof is completed by showing that $I_{\cap }^{mc}\left(X,Y\right)\leq C\left(X\wedge Y\right)$. Construct the bipartite graph with vertex set X∪Y and edges (x,y) if p(x,y)>0. Consider the set of *maximally connected components* $MCC=\{CC_{1},\ldots ,CC_{l}\}$ for some l≥1, where each $CC_{i}$ refers to a maximal set of connected edges. Let $CC_{i},i\leq l$ be an arbitrary set in MCC. Suppose that the edges $\left(x_{1},y_{1}\right)$ and $\left(x_{1},y_{2}\right)$ (with $y_{1}\neq y_{2}$) are in $CC_{i}$. This means that the channels $K^{X}:=K^{X|T}$ and $K^{Y}:=K^{Y|T}$ have rows corresponding to the outcomes $T=(x_{1},y_{1})$ and $T=(x_{1},y_{2})$ of the form

$$K^{X}=\left[\begin{array}{ccccccc} & & & \vdots & & & \\ 0 & \cdots & 0 & 1 & 0 & \cdots & 0\\ 0 & \cdots & 0 & 1 & 0 & \cdots & 0\\ & & & \vdots & & & \end{array}\right],\;K^{Y}=\left[\begin{array}{cccccccc} & & & \vdots & & & & \\ 0 & \cdots & 0 & 1 & 0 & 0 & \cdots & 0\\ 0 & \cdots & 0 & 0 & 1 & 0 & \cdots & 0\\ & & & \vdots & & & & \end{array}\right].$$

Choosing p(t)=[0,…,0,a,1−a,0,…,0], that is, $p(T=\left(x_{1},y_{1}\right))=a$ and $p(T=\left(x_{1},y_{2}\right))=1-a$, we have ∀a∈[0,1],I(X;T)=0, which implies that the solution *Q* must be such that ∀a∈[0,1],I(Q;T)=0 (from the definition of the *more capable* order), which in turn implies that the rows of $K^{Q}$ corresponding to these outcomes must be the same to ensure that they yield I(Q;T)=0 under this set of distributions. We may choose the values of those rows to be the same as those rows from $K^{X}$, that is, a row that is composed of zeros except for one of the positions whenever $T=(x_{1},y_{1})$ or $T=(x_{1},y_{2})$. On the other hand, if the edges $\left(x_{1},y_{1}\right)$ and $\left(x_{2},y_{1}\right)$ (with $x_{1}\neq x_{2}$) are in $CC_{i}$, the same argument leads to the conclusion that the rows of $K^{Q}$ corresponding to the outcomes $T=(x_{1},y_{1})$, $T=(x_{1},y_{2})$ and $T=(x_{2},y_{1})$ must be the same. Applying this argument to every edge in $CC_{i}$, we can conclude that the rows of $K^{Q}$ corresponding to outcomes $\left(x,y\right)\in CC_{i}$ must all be the same. Using this argument for every set $CC_{1},\ldots ,CC_{l}$ implies that if two edges are in the same CC, the corresponding rows of $K^{Q}$ must be the same. These corresponding rows of $K^{Q}$ may vary between different CCs; however, for the same CC they must be the same.

We are left with the choice of appropriate rows of $K^{Q}$ for each corresponding $CC_{i}$. Because I(Q;T) is maximized by a deterministic relation between *Q* and *T* and, as suggested before, we choose a row that is composed of zeros except for one of the positions for each $CC_{i}$ such that *Q* is a deterministic function of *T*, this admissible point *Q* implies that $Q=f_{1}\left(X\right)$ and $Q=f_{2}\left(Y\right)$, as *X* and *Y* are also functions of *T* under the channel perspective. For this choice of rows, we have

$$\begin{array}{ccccc} I_{\cap }^{mc}\left(X,Y\right)\;=\; & \underset{Q}{\sup}I\left(Q;T\right) & \leq & \underset{Q}{\sup}H\left(Q\right) & =\;\;C\left(X\wedge Y\right)\\ & s.t.\;\;Q{⪯}_{mc}X & & s.t.\;\;Q=f_{1}\left(X\right) & \\ & \;Q{⪯}_{mc}Y & & \;Q=f_{2}\left(Y\right) & \end{array}$$

where we have used the fact that I(Q;T)≤min{H(Q),H(T)} to conclude that $I_{\cap }^{mc}\left(X,Y\right)\leq C\left(X\wedge Y\right)$. Hence $I_{\cap }^{ln}\left(X,Y\right)=I_{\cap }^{ds}\left(X,Y\right)=I_{\cap }^{mc}\left(X,Y\right)=C\left(X\wedge Y\right)$ if *T* is a copy of the input.    □

Bertschinger et al. [14] suggested what later became known as the (*) assumption, which states that in the bivariate source case any sensible measure of unique information should only depend on $K^{\left(1\right)},K^{\left(2\right)}$, and p(t). It is not clear that this assumption should hold for every PID. It is trivial to see that all the introduced II measures satisfy the (*) assumption.

We conclude with several applications of the proposed measures to famous (bivariate) PID problems; the results are shown in Table 2. Due to the channel design in these problems, computation of the proposed measures is fairly trivial. We assume that the input variables are binary (taking values in {0,1}), independent, and equiprobable.

We note that in these fairly simple toy distributions all of the introduced measures yield the same value. This is not surprising when the distribution $p(t,y_{1},y_{2})$ yields $K^{\left(1\right)}=K^{\left(2\right)}$, which implies that $I\left(T;Y_{1}\right)=I\left(T;Y_{2}\right)=I_{\cap }^{k}\left(Y_{1},Y_{2}\right)$, where *k* refers to any of the introduced preorders, as is the case in the $T=Y_{1}\;\mathrm{AND}\;Y_{2}$ and $T=Y_{1}+Y_{2}$ examples. Less trivial examples lead to different values over the introduced measures. We present distributions showing that our three introduced measures lead to novel information decompositions by comparing them to the following existing measures: $I_{\cap }^{◃}$ from Griffith et al. [32], $I_{\cap }^{\mathrm{MMI}}$ from Barrett [33], $I_{\cap }^{\mathrm{WB}}$ from Williams and Beer [1], $I_{\cap }^{\mathrm{GH}}$ from Griffith and Ho [38], $I_{\cap }^{\mathrm{Ince}}$ from Ince [20], $I_{\cap }^{\mathrm{FL}}$ from Finn and Lizier [39], $I_{\cap }^{\mathrm{BROJA}}$ from Bertschinger et al. [14], $I_{\cap }^{\mathrm{Harder}}$ from Harder et al. [13], and $I_{\cap }^{\mathrm{dep}}$ from [16]. We used the dit package [40] to compute them, along with the code provided in [21]. Consider counterexample 1 from [22] with p=0.25,ϵ=0.2,δ=0.1, provided by

$$K^{\left(1\right)}=\left[\begin{array}{cc} 0.25 & 0.75\\ 0.35 & 0.65 \end{array}\right],\;K^{\left(2\right)}=\left[\begin{array}{cc} 0.675 & 0.325\\ 0.745 & 0.255 \end{array}\right].$$

These channels satisfy $K^{\left(2\right)}{⪯}_{ln}K^{\left(1\right)}$ and $K_{d}^{\left(2\right)}K^{\left(1\right)}$ from Korner and Marton [22]. This is an example that satisfies $I_{\cap }^{ln}\left(Y_{1},Y_{2}\right)=I\left(T;Y_{2}\right)$ for a given distribution p(t). It is noteworthy to see that even though there is no degradation order between the two channels, we nonetheless have $I_{\cap }^{d}\left(Y_{1},Y_{2}\right)>0$, as there is some non-trivial channel $K^{Q}$ that satisfies $K^{Q}{⪯}_{d}K^{\left(1\right)}$ and $K^{Q}{⪯}_{d}K^{\left(2\right)}$. In Table 3, we present various PIDs under different measures after choosing p(t)=[0.4,0.6] (which yields $I(T;Y_{2})\approx 0.004$) and assuming $p\left(t,y_{1},y_{2}\right)=p\left(t\right)p\left(y_{1}|t\right)p\left(y_{2}|t\right)$.

We write $I_{\cap }^{ds}=$ * here, as we do not yet have a way to find the ‘largest’ *Q* such that $Q{⪯}_{ds}K^{\left(1\right)}$ and $Q{⪯}_{ds}K^{\left(2\right)}$ (see counterexample 2 from [22] for an example of channels $K^{\left(1\right)},K^{\left(2\right)}$ that satisfy $K^{\left(2\right)}{⪯}_{mc}K^{\left(1\right)}$ while $K^{\left(2\right)}{⋠}_{ln}K^{\left(1\right)}$, leading to different values of the proposed II measures). An example of $K^{\left(3\right)},K^{\left(4\right)}$ that satisfy $K^{\left(4\right)}{⪯}_{ds}K^{\left(3\right)}$ while $K^{\left(4\right)}{⋠}_{d}K^{\left(3\right)}$ is presented by (Américo et al. [23], page 10), provided by

$$K^{\left(3\right)}=\left[\begin{array}{cc} 1 & 0\\ 0 & 1\\ 0.5 & 0.5 \end{array}\right],\;K^{\left(4\right)}=\left[\begin{array}{cc} 1 & 0\\ 1 & 0\\ 0.5 & 0.5 \end{array}\right].$$

There is no stochastic matrix $K^{U}$ such that $K^{\left(4\right)}=K^{\left(3\right)}K^{U}$ while $K^{\left(4\right)}{⪯}_{ds}K^{\left(3\right)}$, as $K^{\left(4\right)}={⋄}_{1,2}K^{\left(3\right)}$. Using (10), it is possible to check whether there is any *less noisy* relation between the two channels. (Compute (10) with $V=K^{\left(4\right)},W=K^{\left(3\right)}$, p(t)=[0,0,1], and q(t)=[0.1,0.1,0.8] to conclude that $K^{\left(4\right)}{⋠}_{ln}K^{\left(3\right)}$, then switch the roles of *V* and *W* and set p(t)=[0,1,0] and q(t)=[0.1,0,0.9] to conclude that $K^{\left(3\right)}{⋠}_{ln}K^{\left(4\right)}$). We present the decomposition of $p\left(t,y_{3},y_{4}\right)=p\left(t\right)p\left(y_{3}|t\right)p\left(y_{4}|t\right)$ for the choice of p(t)=[0.3,0.3,0.4] (which yields $I(T;Y_{4})\approx 0.322$) in Table 4.

We write $I_{\cap }^{ln}=0^{*}$ because we conjecture, after numerical experiments based on (10), that the ‘largest’ channel that is less noisy than both $K^{\left(3\right)}$ and $K^{\left(4\right)}$ is a channel that satisfies I(Q;T)=0. (We tested all 3×3 row-stochastic matrices with entries that take values in {0,0.1,0.2,…,0.9,1} with all distributions p(t) and q(t) having entries that take values in the same set.)

## 7. Conclusions and Future Work

In this paper, we have introduced three new measures of *intersection information* for the *partial information decomposition* (PID) framework based on preorders between channels implied by the degradation/Blackwell order. The new measures were obtained from the orders by following the approach recently proposed by Kolchinsky [21]. The main contributions and conclusions of this paper can be summarized as follows:

- We show that a measure of intersection information that satisfies the axioms by Kolchinsky [21] and is based on a preorder, satisfies the Williams–Beer axioms as well [1].
- As a corollary of the previous result, the proposed measures satisfy the Williams–Beer axioms, and can be extended beyond two sources.
- We demonstrate that if there is a degradation ordering between the sources, then the measures coincide in their decomposition. Conversely, if there is no degradation ordering (i.e., only a weaker ordering) between the source variables, the proposed measures lead to novel finer information decompositions that capture different finer information.
- We show that while the proposed measures do not satisfy the *identity property* (IP) [13], they do satisfy the *independent identity property* (IIP) [20].
- We formulate the optimization problems that yield the proposed measures, and derive bounds by relating them to existing measures.

Finally, we believe that this paper opens several avenues for future research; thus, we point to several directions that could be pursued in upcoming work:

- Investigating conditions to verify whether two channels $K^{\left(1\right)}$ and $K^{\left(2\right)}$ satisfy $K^{\left(1\right)}{⪯}_{ds}K^{\left(2\right)}$.
- Kolchinsky [21] showed that when computing $I_{\cap }^{d}\left(Y_{1},\ldots ,Y_{n}\right)$, it is sufficient to consider variables *Q* with a support size of at most $\sum_{i}\left|S_{Y_{i}}\right|-n+1$, which is a consequence of the admissible region of $I_{\cap }^{d}\left(Y_{1},\ldots ,Y_{n}\right)$ being a polytope. The same is not the case with the *less noisy* or the *more capable* measures; hence, it is not clear whether it is sufficient to consider *Q* with the same support size, which could represent a direction for future research.
- Studying the conditions under which different *intersection information* measures are continuous.
- Implementing the introduced measures by addressing their corresponding optimization problems.
- Considering the usual PID framework, except that instead of decomposing I(T;Y)=H(Y)−H(Y|T), where *H* denotes the Shannon entropy, other mutual informations induced by different entropy measures could be considered, such as the *guessing entropy* [41] or the *Tsallis entropy* [42] (see the work of Américo et al. [23] for other core-concave entropies that may be decomposed under the introduced preorders, as these entropies are consistent with the introduced orders).
- Another line for future work might be to define measures of union information using the introduced preorders, as suggested by Kolchinsky [21], and to study their properties.
- As a more long-term research direction, it would be interesting to study how the approach taken in this paper can be extended to quantum information; the fact that partial quantum information can be negative might open up new possibilities or create novel difficulties [43].

## Figures and Tables

**Table 1:** Copy distribution.

T	$Y_{1}$	$Y_{2}$	$p(t,y_{1},y_{2})$
(0, 0)	0	0	p(T=(0,0))
(0, 1)	0	1	p(T=(0,1))
(1, 0)	1	0	p(T=(1,0))
(1, 1)	1	1	p(T=(1,1))

**Table 2:** Application of the proposed measures to famous PID problems.

Target	$I_{\cap }^{◃}$	$I_{\cap }^{d}$	$I_{\cap }^{\ln}$	$I_{\cap }^{\mathrm{ds}}$	$I_{\cap }^{\mathrm{mc}}$	$I_{\cap }^{\mathrm{MMI}}$
$T\;=\;Y_{1}\;\mathrm{AND}\;Y_{2}$	0	0.311	0.311	0.311	0.311	0.311
$T\;=\;Y_{1}\;+\;Y_{2}$	0	0.5	0.5	0.5	0.5	0.5
$T=Y_{1}$	0	0	0	0	0	0
$T\;=\;(Y_{1},Y_{2})$	0	0	0	0	0	1

**Table 3:** Different decompositions of $p(t,y_{1},y_{2})$.

$I_{\cap }^{◃}$	$I_{\cap }^{d}$	$I_{\cap }^{\ln}$	$I_{\cap }^{\mathrm{ds}}$	$I_{\cap }^{\mathrm{mc}}$	$I_{\cap }^{\mathrm{MMI}}$	$I_{\cap }^{\mathrm{WB}}$	$I_{\cap }^{\mathrm{GH}}$	$I_{\cap }^{\mathrm{Ince}}$	$I_{\cap }^{\mathrm{FL}}$	$I_{\cap }^{\mathrm{BROJA}}$	$I_{\cap }^{\mathrm{Harder}}$	$I_{\cap }^{\mathrm{dep}}$
0	0.002	0.004	*	0.004	0.004	0.004	0.002	0.003	0.047	0.003	0.004	0

**Table 4:** Different decompositions of $p(t,y_{3},y_{4})$.

$I_{\cap }^{◃}$	$I_{\cap }^{d}$	$I_{\cap }^{\ln}$	$I_{\cap }^{\mathrm{ds}}$	$I_{\cap }^{\mathrm{mc}}$	$I_{\cap }^{\mathrm{MMI}}$	$I_{\cap }^{\mathrm{WB}}$	$I_{\cap }^{\mathrm{GH}}$	$I_{\cap }^{\mathrm{Ince}}$	$I_{\cap }^{\mathrm{FL}}$	$I_{\cap }^{\mathrm{BROJA}}$	$I_{\cap }^{\mathrm{Harder}}$	$I_{\cap }^{\mathrm{dep}}$
0	0	$0^{*}$	0.322	0.322	0.322	0.193	0	0	0.058	0	0	0

## Data Availability

Not applicable.
